# Community-friendly diagnostics: Who are tests for?

**DOI:** 10.1371/journal.pgph.0002915

**Published:** 2024-03-11

**Authors:** Fifa A. Rahman, Kenly Sikwese

**Affiliations:** 1 Matahari Global Solutions, Kuala Lumpur, Malaysia; 2 Afrocab Treatment Access Partnership, Lusaka, Zambia; PLOS: Public Library of Science, UNITED STATES; McGill University, CANADA

While vaccine inequity during the COVID-19 pandemic received widespread attention, less attention has been paid to testing inequities during the pandemic. The pandemic saw deployment of both PCR and rapid testing programs that were not accessible for communities in many low-income countries, especially the African region. For example, in Nigeria, Africa’s most populous country with 200 million people, only 3 million tests had been carried out by November 2021 [1]. During the first wave of COVID-19 infections In the Democratic Republic of Congo (DRC), only 0.3 tests per 10,000 people were being carried out in contrast with the minimum recommended amount of 10 tests per 10,000 people [2]. Even before the pandemic, the Lancet Commission on Diagnostics estimated that nearly half the world’s population has little to no access to diagnostics [3].

## What obstacles exist for communities trying to access testing?

In Somalia, for example, where 25–30% of the population are nomadic, PCR tests were often rolled out knowing that no address could be registered for many people and that results would not be able to be returned to them [4]. In the Nyiragongo health zone in North Kivu, Democratic Republic of the Congo, amidst challenging environments with the M23 rebellion and high levels of internal displacement, samples needed to be sent 19 kilometers south to Goma for analysis, and results would often come back two weeks after the patient had already been discharged [4]. These delayed turnaround times were attributed to PCR analysis for COVID-19 only provided by a few laboratories in cities [2].

In Uganda, an assessment conducted among fishing communities and people with disabilities showed that COVID-19 information was often disseminated in English rather than in local languages such as Luganda and Swahili–meaning communities were often left out [5]. The same assessment showed insufficient health workers to meet demand, long lines and waiting times, and poor roads, long distances, or expensive transport costs that represented significant barriers to care [5]. People with disabilities and mobility challenges were unable to travel to get PCR testing or subsequent treatment services [5]. Research shows that over 1.3 million tests were conducted between March 2020 and June 2021 in Uganda for a population of 47.7 million [6]. According to officials, detected cases were likely only 10–15% of the true number of COVID-19 infections, signifying that there was undertesting [7].

An assessment in Madagascar focusing on access to ten essential diagnostics for primary care settings found that individuals often had to travel an average of 5.5 kilometres by foot and by bus to access a testing facility [8]. Given that the average cost of a bus ride constitutes 42% of an individual’s daily income, this presents significant financial barriers to care [9]. There is also poor road infrastructure, so many had to travel by foot through rice fields and uneven terrain to get to healthcare centres [9].

This leads to questions of:

1. **Who are tests for**? Are they predominantly for governments to ascertain how quickly a pathogen is spreading? Or are they predominantly so that communities can know their status? How can we work to ensure that both objectives are achieved but that we prioritise the right of people to know their status–and seek subsequent treatment services as they are needed?
2. **How do we create community-friendly diagnostics services?** How can we listen to what communities have reported, such as in the assessments described above, and adapt testing and treatment services to meet people where they are? How can we transform diagnostics to truly serve the needs of the people that need them the most?

**Fig 1:**
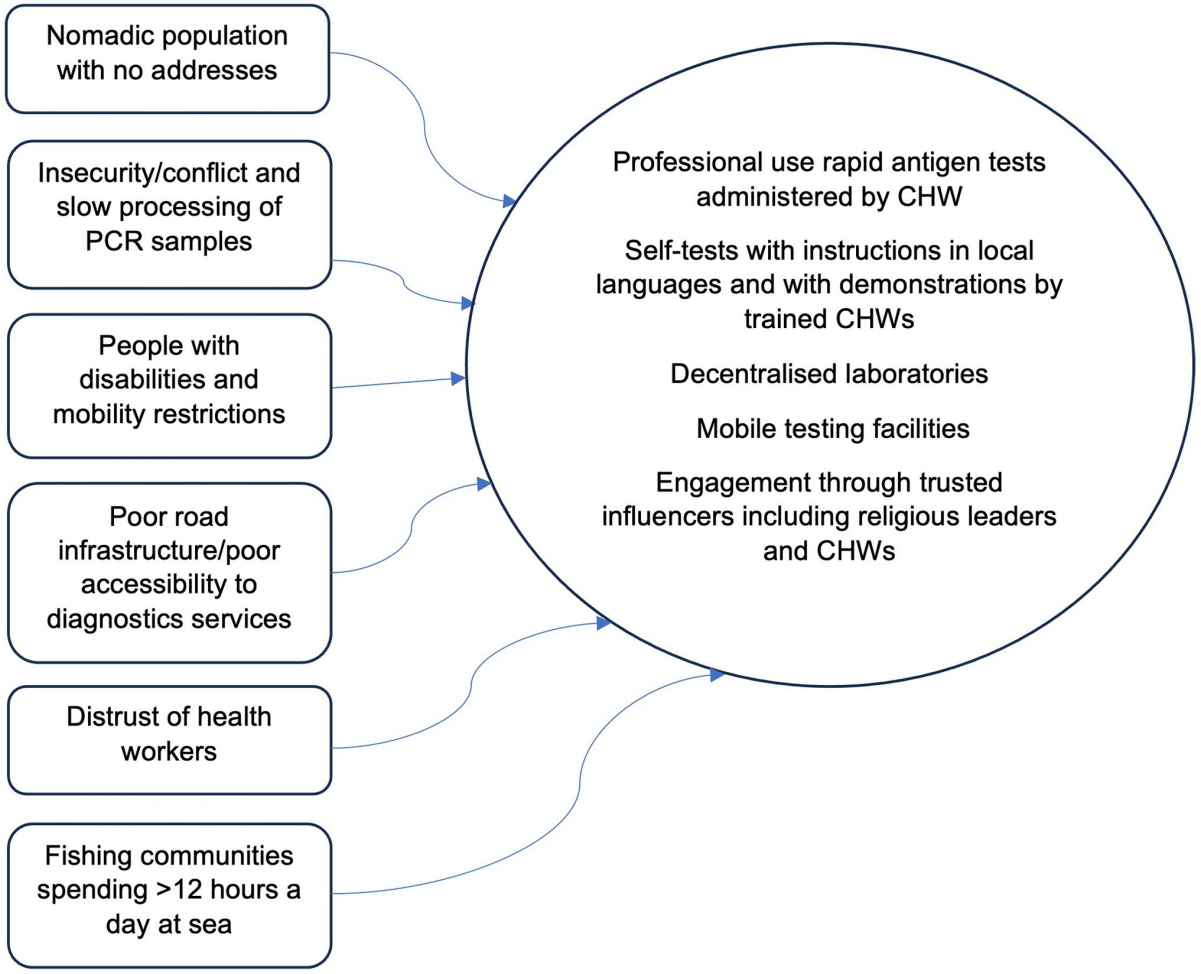
Factors to consider in the design of community-friendly diagnostics programmes.

Fig 1 below offers an illustration of how we can better service communities and what best practice could look like.

## Incorporating a intersectional equity lens at the outset of diagnostics plans

There are countless other examples of communities that we have not included here–but what this diagram illustrates is that for the right to health vis-à-vis diagnostics to be truly fulfilled, we need three core components guiding diagnostics deployment:

1. A **meaningful understanding of communities**, including their strengths and routines, their challenges and vulnerabilities, and what their preferred access routes (or care seeking patterns) are.
2. **Pragmatism** in response/diagnostics teams. If PCR facilities in their current locations won’t help increase access–be pragmatic and innovative.
3. That the **right to know one’s status is equally if not more important** than the desire of governments to conduct surveillance or impose public health measures such as quarantine.

Global policy factors also influence access to diagnostics for communities. For example, UNICEF and Global Fund required WHO guidance to procure COVID-19 self-tests for deployment. Unfortunately, the issuance of self-testing guidelines were delayed to March 2023, beyond the acute phase of the pandemic, because of an assumption that self-tests would not lead to individuals ‘linking to public health action’, i.e., either seeking treatment or self-isolation [10]. While self-testing options were rolled out quickly in high-income countries, access to self-testing for LMICs was stymied in part by these delays. As a result, large procurers were not able to deploy self-tests until it was too late–despite strong research showing high acceptability of self-tests for COVID-19 in multiple African countries [11].

Intellectual property (IP) policies also affect community accessibility to diagnostics. For example, GeneXpert machines are well-distributed within countries due to their use in TB programmes, however IP policies and long patents result in very expensive cartridges and reagents. Local, less expensive production of these diagnostic tools, which would go a long way towards increasing community-level accessibility, is currently prevented by IP protections [12].

Community-friendly diagnostics policies in future pandemics therefore will need to be supported by more agile, regional mechanisms and guidelines rather than waiting for inefficient and maladapted processes at the global level.

LMICs will need to take responsibility for the health of their citizens and not be overdependent on systems that may not be available during a pandemic [13]. Agile decision-making mechanisms by Ministries of Health in the absence of WHO normative guidance is necessary. LMICs, especially the Africa region, must also invest in regional manufacturing of diagnostics, to reduce excessive reliance on imported and expensive products.

A report from the International Pandemic Preparedness Secretariat and FIND emphasised the right of individuals to know their status [14]. The report further emphasises that as regards self-testing, this need not be isolated from wider systems–and could be linked to digital technologies to ensure better linkage to clinical care [15] and could be well-integrated into community systems. The successful roll-out of HIV self-testing in the African region clearly demonstrates the value of expanding self-testing to other conditions [15, 16].

These examples and more highlight how we need to do so much more to ensure that diagnostics are community friendly. With a little dose of pragmatism, emphasis on the right to know one’s status and a reminder of who these tests are for, as well as a genuine effort to understand the communities we are serving, we can design diagnostics systems that are truly community friendly and are aimed towards equity.
